# Does cardiac rehabilitation meet minimum standards: an observational study using UK national audit?

**DOI:** 10.1136/openhrt-2016-000519

**Published:** 2017-01-09

**Authors:** Patrick Doherty, Ahmad Salman, Gill Furze, Hasnain M Dalal, Alexander Harrison

**Affiliations:** 1 Department of Health Sciences, University of York, York, North Yorkshire, UK; 2 Centre for Technology Enabled Health Research, Coventry University, Coventry, West Midlands, UK; 3 University of Exeter Medical School (Truro Campus), Knowledge Spa, Royal Cornwall, Truro, UK

**Keywords:** CORONARY ARTERY DISEASE

## Abstract

**Objective:**

To assess the extent by which programmes meet national minimum standards for the delivery of cardiac rehabilitation (CR) as part of the National Certification Programme for Cardiovascular Rehabilitation (NCP_CR).

**Methods:**

The analysis used UK National Audit of Cardiac Rehabilitation (NACR) data extracted and validated for the period 2013–2014 set against six NCP_CR measures deemed as important for the delivery of high-quality CR programmes. Each programme that achieved a single minimum standard was given a score of 1. The range of the scoring for meeting the minimum standards is between 1 and 6. The performance of CR programmes was categorised into three groups: high (score of 5–6), middle (scores of 3–4) and low (scores of 1–2). If a programme did not meet any of the six criteria, they were considered to have failed.

**Results:**

Data from 170 CR programmes revealed statistically significant differences among UK CR programmes. The principal findings were that, based on NCP_CR criteria, 30.6% were assessed as high performance with 45.9% as mid-level performance programmes, 18.2% were in the lower-level and 5.3% failed to meet any of the minimum criteria.

**Conclusions:**

This study shows that high levels of performance is achievable in the era of modern cardiology and that many CR programmes are close to meeting high performance standards. However, substantial variation, below the recommended minimum standards, exists throughout the UK. National certification should be seen as a positive step to ensure that patients, irrespective of where they live, are accessing quality services.

**Key questions**

**What is already known about this subject?**

- Recent clinical review of cardiac rehabilitation (CR) highlights that CR is highly effective but warns that not all programmes are working to the minimum standards.

**What does this study add?**

- This is the first study in the UK identifying the proportion of programmes meeting national minimum standards for the delivery of CR. Only 30% of the UK CR programmes met the criteria for high performance CR. This study is the first to evaluate CR against minimum standards and report the extent of deficit in UK CR services.

**How might this impact on clinical practice?**

- This paper shows that high performance is achievable in the modern cardiology era and that many other programmes deemed as being midlevel performance are close to meeting high performance standards. It has also shown that the National Certification Programme for Cardiovascular Rehabilitation (NCP_CR) criteria are able to differentiate the quality of CR delivery.

## Introduction

Cardiovascular disease (CVD) is the number one cause of death that is globally responsible for an estimated 17.5 million people deaths, 31% of all global deaths in 2012.^1^ In 2014, CVD caused 27% of all deaths in the UK.^2^ On the basis of international guidelines, underpinned by Class I evidence, cardiac rehabilitation (CR) is recommended as an effective intervention for patients diagnosed with CVDs.^3–6^ CR is defined as a structured, multicomponent, tailored intervention that is delivered by a skilled multidisciplinary team.^5^ ^6^ The British Association for Cardiovascular Prevention and Rehabilitation (BACPR) recommended minimum standards, National Institute for Health and Care Excellence (NICE) clinical guidance and the National Certification Programme for CR (NCP_CR) seek to ensure that routine provision of CR programmes closely resembles that delivered by effective clinical trials.^4^ ^7–9^ The National Audit of Cardiac Rehabilitation (NACR), funded by the British Heart Foundation, is a clinical audit that monitors CR services in the UK in terms of service delivery and patient outcome.^10^ According to the 2015 NACR report, the number of CR programmes delivering core CR in 2013–2014 was 308.^10^ Numerous clinical trials and systematic reviews have shown the effectiveness of CR over the last 20 years.^3^ ^11^ The updated Cochrane review reported that CR is proved to reduce cardiovascular mortality, hospital admissions in addition to improving health-related quality of life.^3^ On the other hand, the conclusion from the largest UK-based randomised controlled trial ‘Rehabilitation after myocardial infarction trial (RAMIT)’ of comprehensive CR in the modern era of medical management showed that CR does not reduce mortality or morbidity and has no beneficial effect on psychosocial well-being or lifestyle.^12^ RAMIT was included in the Anderson *et al*^3^ review alongside 62 other trials and did not alter the overall cardiovascular mortality benefit. The negative results of RAMIT appear to differ from those of the latest Cochrane reviews. The negative findings of this trial have also led to scepticism about the delivery of UK-based CR programmes.^13^ ^14^ Moreover, a recent clinical review of CR published in the *British Medical Journal* highlights that CR is highly effective but warns that not all programmes are working to the minimum standards.^11^ The NACR is committed to promoting and supporting quality service provision based on measurable indicators of successful delivery. The aim of this study was to assess the extent by which programmes meet national minimum standards for the delivery of CR.

## Methods

### Data collection

The analyses were conducted using individual patient data collected electronically by the UK NACR which has approval to collect anonymised patient data for a range of clinical variables.^15^ Data are collected under 251 approvals that are reviewed annually by the Health and Social Care information Centre (HSCIC). The audit is voluntary, collecting local programme-level data on the delivery of CR alongside patient-level data on patients who undergo CR in the UK, including details of the initiating event, treatment type, risk factors, medication, patient demographics and pre-CR clinical outcomes and post-CR clinical outcomes. The data from 1 April 2013 to 31 March 2014, which relates to the first year of the NCP_CR minimum standards, have been validated and extracted to support this analysis. Patients were included in the analyses if they started CR, had been assessed at baseline and had follow-up data at an assessment post-CR. This observational study was reported following the guidelines of the Strengthening the Reporting of Observational Studies in Epidemiology (STROBE).

### Service delivery measures

The BACPR-NACR National Certification Programme for Cardiovascular Rehabilitation (NCP_CR) aims to achieve a minimum level of service delivery across the UK and has clear guidance (available by emailing: education@bacpr.com) which is based on NACR patient-level and programme-level data extracted as the NCP_CR report.^9^ The latter was used in this study to assess whether a CR programme met the minimum service delivery standards. Within the NCP_CR report, six field measures, deemed as important for defining the delivery of high-performance CR programmes, were used alongside 95% CI as the part of certification criteria derived from all three countries (England, Wales and Northern Ireland). The NCP_CR minimum service delivery criteria used to define high-performance CR programmes was based on NICE guidance and national UK CR statistics (NACR 2015 report). The criteria included:

- offered to all priority groups (PG):
  Myocardial infarction (MI)
  Percutaneous coronary intervention (PCI)
  Coronary artery bypass surgery (CABG)
  Heart failure (HF)
- ≥69% of core CR patients with recorded assessment before starting formal CR programme (ax1)
- ≥49% of core CR patients (end of CR) with recorded assessment after completing CR programme (ax2)
- Median waiting time from referral to start (TRS) of CR—MI/PCI (TRS_CR/MIPCI) was within 40 days
- Median waiting time from referral to start of CR—CABG (TRS_CR/CABG) was within 54 days
- Median duration of CR programmes was 54 days for conventional delivery or 42 days where the Heart Manual (an evidence-based 6-week facilitated self-management programme) was the sole method of delivery.^16^ ^17^

### NCP_CR scoring

Each programme that achieved a single minimum standard was given a score of 1. The range of the scores is between 1 and 6. The performance of CR programmes was categorised into three groups: high (scores of 5–6), middle (scores of 3–4) and low (scores of 1–2). If a programme did not meet any of the six criteria, they were considered to have failed.

### Statistical analysis

The analyses were conducted using IBM Statistical Package for Social Sciences (SPSS) software statistics V.23 (SPSS, Chicago, Illinois, USA). Analyses were conducted using all available data from CR programme centres across the UK, to minimise selection bias. Programmes have been aggregated to identify those who met the minimum criteria. Mean and frequency tables were generated to score the programmes according to the certification categories. A χ^2^ test for association was conducted between meeting each minimum standard and where the programme sat in the performance group. Data were analysed by using one-way analysis of variance (ANOVA) test, which was conducted to determine whether the minimum criteria were different among performance groups. Games-Howell method was conducted while performing ANOVA for multiple comparisons. Partial η^2^ have been reported as an effect size. A value of p≤0.05 was considered statistically significant.

## Results

The analysis was derived from 170 CR programmes in the UK, of which 52 (30.6%) scored 5 or 6, so making them high-performance programmes. Middle performance programmes being the largest group accounting for 78 programmes (45.9%). However, 31 programmes (18.2%) were considered as low-performance programmes. Programme performance categories are presented in table 1.

**Table 1:** Programme performance categories

Programme performance rating	Frequency	Percentage
Poor	9	5.3
Low	31	18.2
Middle	78	45.9
High	52	30.6

15.9% was the percentage of programmes (27 programmes) who met all the minimum criteria. 84.1% of the programmes offering CR were below the scores required for meeting all minimum criteria.

**Figure 1:**
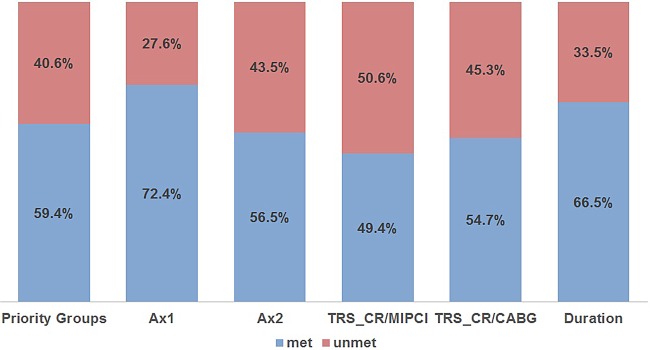
Percentage of total CR programmes meeting and not meeting each of the six fields of the minimum criteria. CR, cardiac rehabilitation.

The percentage of programmes that met each specific criterion is presented in figure 1.

Assessment 1 (ax1) was the largest percentage meeting field (72.4%) on the criteria while waiting time from referral to start (TRS) of CR—MI/PCI (TRS_CR/MIPCI) was the smallest percentage meeting field (49.4%).

The extent by which CR programmes met each minimum standard among performance categories varied significantly (table 2). Ax1 is the highest minimum standard met among the low (51.6%), middle (71.8%) and high (98.1%) performance programmes. On the other hand, the lowest minimum performance category was for the types of patient priority groups included (9.7%), TRS_CR/MIPCI (43.6%) and Ax2 (84.6%) among low-performance, middle performance and high-performance programmes, respectively.

**Table 2:** Frequency and percentage of each minimum standard among performance categories

Minimum standard	Low (31)	Middle (78)	High (52)	Cramer's V
PG	3 (9.7%)	48 (61.5%)	50 (96.2%)	0.62*
Ax1	16 (51.6%)	56 (71.8%)	51 (98.1%)	0.39*
Ax2	7 (22.6%)	45 (57.7%)	44 (84.6%)	0.44*
TRS_CR/MIPCI	4 (12.9%)	34 (43.6%)	46 (88.5%)	0.55*
TRS_CR/CABG	7 (22.6%)	38 (48.7%)	48 (92.3%)	0.52*
Duration	14 (45.2%)	51 (65.4%)	48 (92.3%)	0.37*

Ax1, assessment 1; Ax2, assessment 2; PG, priority group; TRS_CR/CABG, median waiting time from referral to start of CR—CABG; TRS_CR/MIPCI, median waiting time from referral to start (TRS) of CR—MI/PCI.

*p<0.001.

A χ^2^ test for association was conducted between meeting each minimum standard and the three performance categories. All expected cell frequencies were >5. There was a statistically significant association between meeting each standard and performance categories, p<0.001 at all. There was moderate to strong association between meeting each standard and performance categories (table 2). The PG standard among performance categories had the largest association (φ=0.62) while the duration of CR programme standard had the lowest association among all categories (φ=0.37).

A one-way ANOVA was conducted to determine whether the mean value of each of the five fields of the criteria (the five fields: % of ax1, % of ax2, median waiting TRS_CR/MIPCI, median waiting TRS_CR/CABG and median duration) were different among performance categories. Table 3 shows that the average of the standards in the low-performance programmes was statistically and significantly different to either the middle performance or high-performance programmes. When comparing the average standards in each group, every standard in the low-performance programmes was outside the criteria. This differed to the middle performance programmes, where some standards were met such as the assessments but both referral times were outside of the boundaries. The high-performance programmes averages all sat within the boundaries.

**Table 3:** ANOVA with post hoc results among performance categories

Minimum standard	Low (26)	Middle (78)	High (52)	(Sig.)	Effect Size
Ax1	68.45%*	76.42%†	89.44%*,†	0.000	0.09
Ax2	41.05%*	52.25%†	63.98%*,†	0.001	0.09
TRS_CR/MIPCI	54.39‡	42.94‡	31.32‡	0.000	0.19
TRS_CR/CABG	61.85*	55.61†	41.99*,†	0.000	0.12
Duration	57.59*	64.56	70.33*	0.031	0.04

Ax1, assessment 1; Ax2, assessment 2; TRS_CR/CABG, median waiting time from referral to start of CR—CABG; TRS_CR/MIPCI, median waiting time from referral to start (TRS) of CR—MI/PCI.

*Post hoc significance between low-performance and high-performance groups, p≤0.05.

†Post hoc significance between middle performance and high-performance groups, p≤0.05.

‡Post hoc significance among low-performance, middle  performance and high-performance groups, p≤0.05.

The effect sizes (partial η^2^) were largest for median waiting time from referral to start CR programme for MIPCI (TRS_CR/MIPCI) and CABG (TRS_CR/CABG) (0.19 and 0.12, respectively) while duration had the lowest effect size (0.04).

## Discussion

There were 170 CR programmes pooled from the patient-level NACR data to identify those who met the minimum standards of the NCP_CR. Statistically significant differences were found among UK CR programmes regarding meeting the minimum standards in terms of delivery of CR in the UK. The principal finding of this study was that, based on the NACR data from 2013 to 2014, only 15.9% (27 programmes out of 170 UK CR programmes) met all the minimum standards included in the NCP_CR report.^10^ This result depends on the use of the more lenient interpretation of the report, where we used the 95% CI of the annual averages of the minimum standards. Using the 95% CI increases the data range for meeting a particular minimum standard. Previously, CR programmes were required to meet a particular data cut-point for the majority of the standards within the NCP_CR report. If this latter method had still been in place, fewer programmes would be classed as high performance. This finding agrees with the warning, given in the recent clinical review of CR published in the *British Medical Journal*, that not all CR programmes are working to the minimum standards.^11^ The results of this study demonstrate the huge variation in meeting the minimum standards among CR programmes. Also, the analysis showed that, within low-performance groups, CR is being delivered later than recommended, not offered for the PG, not underpinned by preassessment and postassessment and is shorter in duration than the recommended minimum standards suggested by the BACPR, NICE service CR commissioning guide and NICE clinical guidance 172.^4^ ^7^ ^8^ Our analysis showed that a large proportion of the variance in the performance groups (38.44% and 19%, respectively) was associated with the minimum standards for offering CR to PG, and with the time from referral to CR start among MI/PCI patients. Despite having tariff-based National Health Service funding and NICE clinical guidelines which define the service specification for the delivery of CR, this study showed that the performance of programmes in the UK varies significantly in terms of meeting the recommended minimum standards. This study is the only UK-specific study that identifies the proportion of programmes meeting national minimum standards for the delivery of CR. This study accounted for six service indicator measures that form part of the NCP_CR report.

This paper shows that high performance is achievable in the modern cardiology era and that many other programmes deemed as being midlevel performance are close to meeting high performance standards. However, substantial unacceptable variation, below the accepted minimum standards, exists. This paper has shown that NCP_CR criteria are able to differentiate the quality of CR delivery and our findings thus support national certification is a positive step to ensuring that patients, irrespective of where they live, are accessing quality services.

## Limitations

The use of an observational approach based on routinely collected patient data is a strength in respect of showing what happens in the real-world, but retrospective observational studies have known limitations in terms of data capture and quality. There are 308 CR programmes in the UK, according to the 2015 NACR report, but only 170 programmes entered NACR data electronically, which was a requirement of this study. Although it can be argued that there are enough data to carry out the analysis, future work should aim to achieve greater capture of available data across the UK. Although CR programmes are encouraged to provide complete patient records, it was expected that a proportion of patient data would be missing due to non-completion of patient records. On the basis of  the NACR data, of all patients who completed CR, 32% did not have a post-CR assessment recorded, which might have affected the representativeness of our sample.

## Conclusions

This study aimed to identify the proportion of programmes meeting national minimum standards for the delivery of CR. Only 30% of the UK CR programmes met the criteria for high-performance CR with a further 18% seen as low performance and 5% failed to meet any of the criteria. This study is the first to evaluate CR against minimum standards and report the extent of deficit in UK CR services. Further research is required to investigate the extent of patient outcomes between high-performance, middle performance and low-performance CR programmes.
